# Correction to: Open chromatin analysis in *Trypanosoma cruzi* life forms highlights critical differences in genomic compartments and developmental regulation at tDNA loci

**DOI:** 10.1186/s13072-022-00459-2

**Published:** 2022-07-06

**Authors:** Alex Ranieri Jerônimo Lima, Herbert Guimarães de Sousa Silva, Saloe Bispo Poubel, Juliana Nunes Rosón, Loyze Paola Oliveira de Lima, Héllida Marina Costa-Silva, Camila Silva Gonçalves, Pedro A. F. Galante, Fabiola Holetz, Maria Cristina Machado Motta, Ariel M. Silber, M. Carolina Elias, Julia Pinheiro Chagas da Cunha

**Affiliations:** 1 Laboratório de Ciclo Celular, Instituto Butantan, São Paulo, SP Brazil; 2 Centro de Toxinas, Resposta Imune E Sinalização Celular (CeTICS), Instituto Butantan, São Paulo, Brazil; 3 Departamento de Microbiologia, Universidade Federal de São Paulo, Escola Paulista de Medicina, Imunologia E Parasitologia, São Paulo, SP Brazil; 4 Laboratório de Ultraestrutura Celular Hertha Meyer, Instituto de Biofísica Carlos Chagas Filho, Universidade Federal Do Rio de Janeiro, IBCCF, CCS, UFRJ, Cidade Universitária, Rio de Janeiro, RJ Brazil; 5 Centro Nacional de Biologia Estrutural E Bioimagem, Rio de Janeiro, RJ Brazil; 6 Centro de Oncologia Molecular, Hospital Sírio Libanês, São Paulo, SP Brazil; 7 Instituto Carlos Chagas, Fiocruz, Curitiba, PR Brazil; 8 Universidade de São Paulo, São Paulo, SP Brazil

## Correction to: Epigenetics & Chromatin (2022) 15:22 https://doi.org/10.1186/s13072-022-00450-x

Following publication of the original article [1], error was identified in author name. The author name Maria Cristina Machado Motta was incorrectly written as Maria Cristina Machado M. Motta.

The Original article has been corrected.
